# THz spectroscopy and deviation from Drude model of LT-GaAs thin films with sub-picosecond charge-carrier lifetime

**DOI:** 10.1039/d6fd00013d

**Published:** 2026-07-10

**Authors:** Jasmin-Clara Bürger, James Normansell, Lianhe Li, Kun Peng, Joshua Freeman, Michael B. Johnston

**Affiliations:** a Department of Physics, University of Oxford, Clarendon Laboratory Parks Road Oxford OX1 3PU UK jasmin.buerger@physics.ox.ac.uk michael.johnston@physics.ox.ac.uk; b School of Electronic and Electrical Engineering, University of Leeds Leeds LS2 9JT UK

## Abstract

High-performance ultrafast terahertz devices such as modulators, photoconductive emitters, and detectors rely on an ultrafast change in conductivity. Semiconductors are suitable for this purpose since their charge-carrier density can be varied by external factors, such as incident photons or an applied electrical potential. High-performance ultrafast terahertz devices require semiconductors with high charge-carrier mobility but low charge-carrier lifetimes. The trade-off between charge-carrier mobility and the short charge-carrier lifetimes of standard semiconductors limits the signal-to-noise ratio of current terahertz devices. Hence, it is essential to develop high-performance materials to overcome these limitations and enable ultrafast terahertz technology. Low-temperature GaAs (LT-GaAs) has shown excellent performance in overcoming this limitation by providing a high charge-carrier mobility and, at the same time, a low charge-carrier lifetime. In this study, we have conducted terahertz time-domain spectroscopy (THz-TDS) and terahertz optical-pump terahertz-probe spectroscopy (OPTPS) on 2 µm-thick LT-GaAs thin films bonded with benzocyclobutene (BCB) to sapphire substrates. With these techniques, we demonstrate excellent sub-picosecond charge-carrier lifetimes across different infrared pump beam fluences. The measured samples showed a charge-carrier lifetime in LT-GaAs of 0.46 ps. We discuss observed saturation effects and how they can influence the shape of the photoconductivity spectrum. We explain the origin of this effect in the context of the experimental technique and charge-carrier dynamics in the material.

## Abstract 2

Fluence-dependent analysis of LT-GaAs with charge-carrier lifetimes of 0.4 ps by optical-pump terahertz-probe spectroscopy and discussion of observed saturation effects.

## Graphical abstract

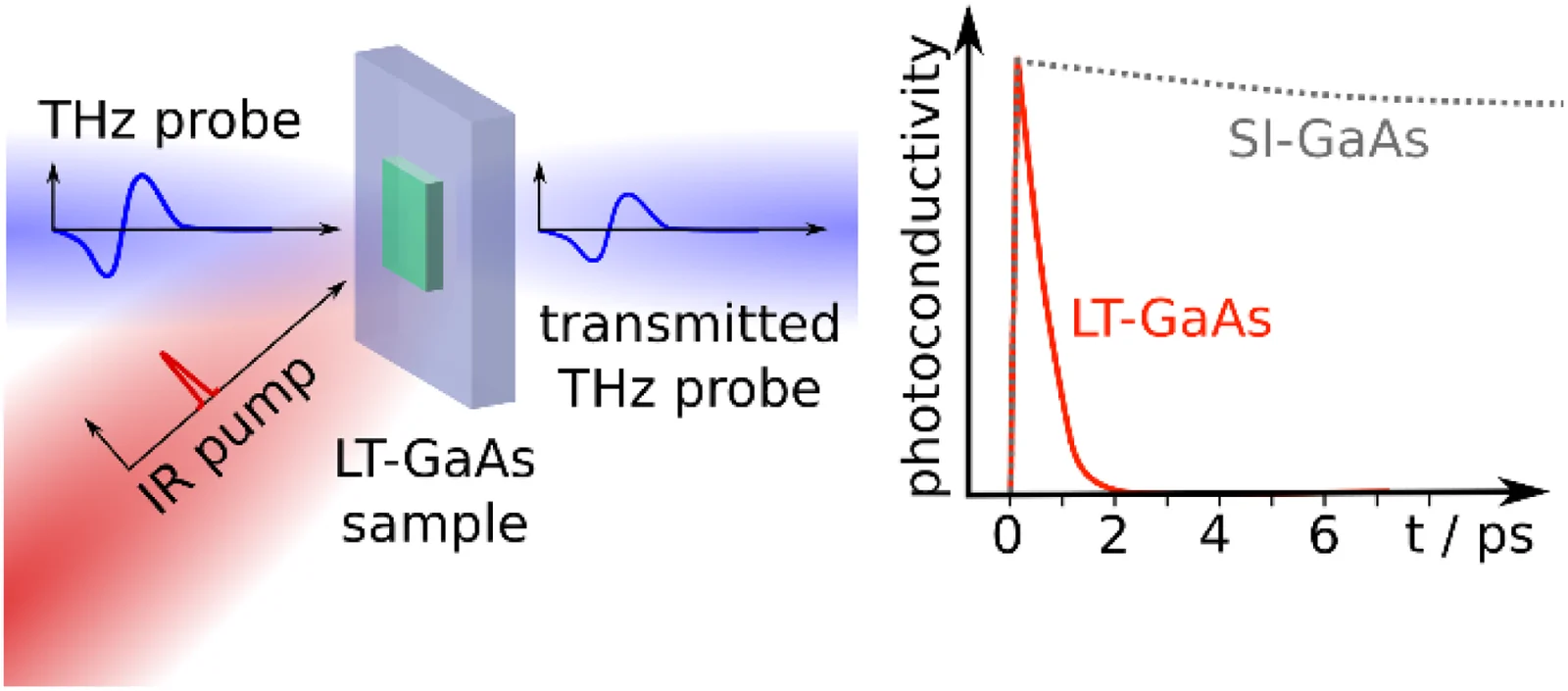

## Introduction

The further increase in the speed of ultrafast terahertz devices with broadband applications depends on the development and characterisation of semiconductors with ultrafast charge-carrier dynamics.^1–3^ Here, the ultrafast charge-carrier dynamics of these materials are outstanding as they allow for quick change of their charge-carrier density controlled by incident photons or an electrical bias voltage.^4–6^ By photoexcitation or an electric bias, additional charge carriers are generated.^1^ Current photoconductive devices, however, are limited by their response time and signal-to-noise ratio.^1,2,7,8^ Both can be improved if the charge carriers in the semiconductor are trapped within a short time.^1^ Hence, high-performance semiconductors, which are promising for optical terahertz devices, require at the same time a short charge-carrier lifetime and a high charge-carrier mobility.^9–11^ In addition, it is essential to determine the charge-carrier concentration for these materials.^12^

Developing semiconductors with this outstanding performance carries challenges for their performance-oriented fabrication and precise time-resolved characterisation.^6,13^ GaAs or other III–V compound semiconductors that have direct band gaps and bandgap energies suitable for photoexcitation with infrared femtosecond lasers, are highly promising to be used as active elements in terahertz devices.^6,13,14^ III–V semiconductors, such as semi-insulating GaAs (SI-GaAs), are generally grown by molecular beam epitaxy (MBE) or metal organic-chemical vapor deposition (MOCVD) at temperatures above 500 °C.^13,15,16^ SI-GaAs has a high charge-carrier mobility, but still has charge-carrier lifetimes in the range >>100 ps.^13,15^ To shorten the charge-carrier lifetime, additional trapping and recombination centres need to be integrated into the crystal lattice.^13,17^ To reach the performance required, two methods are widely studied in the literature, which are (I) low temperature growth of the semiconductor and (II) doping of the semiconductor.^13^ If the GaAs is grown at temperatures between 180 °C and 350 °C, the required defect centres can be integrated into the crystal lattice.^1,13,18^ GaAs grown at such a temperature is called low-temperature-grown GaAs (LT-GaAs).^1,13^ After the epitaxy, a post-annealing step in the range of 600 °C is required to allow defects to recombine, diffuse, or annihilate and thus increased dark resisitivity.^13,18–20^ The defect centres in LT-GaAs are based on excess arsenic, which can reach 1.5 at%.^1,16,19,20^ The excess arsenic forms As antisites, As interstitials and As nanoclusters.^17–19^ Additional defects formed in LT-GaAs are Ga-related vacancies.^17^ The defects are known to function as buried Schottky barriers, which form deep-level bands in the bandgap and lead to significantly reduced charge-carrier lifetimes.^18^ Well-optimised LT-GaAs can achieve the properties of a picosecond and sub-picosecond charge-carrier lifetime,^16^ a low dark current^17^ (resistivity 10^7^ Ω cm) and a high electron mobility^16^ (up to 2000 cm^2^ V^−1^ s^−1^), which are favoured for ultrafast terahertz devices.^13,19^ These properties make LT-GaAs an excellent active material for photoconductive terahertz emitters and detectors with a low signal-to-noise ratio.^7,11^ An alternative method is the doping of the GaAs by ion-implantation with, *e.g.*, arsenic atoms and subsequent annealing.^13,19^

The discussed material properties can be probed and investigated by terahertz spectroscopy techniques.^12,13^ The application of ultrashort terahertz pulses allows for the simultaneous measurement of a wide range of frequencies in the terahertz spectrum.^21^ In particular, optical-pump terahertz-probe spectroscopy (OPTPS) is a powerful tool for studying charge-carrier dynamics in the picosecond range.^2^ OPTPS measures changes in the transmission and reflection of the terahertz probe pulse through a sample after excitation with an optical pump beam.^13,22^ Typically, the THz transmission is significantly reduced within 100 fs of exciting the sample with the pump pulse, followed by a transient increase in THz transmission in the time frame of several 100 ps, depending on the material properties.^15,22^ These behaviours and responses are based on the generation of charge carriers and the filling of the conduction and valence bands.^22^ Hence, by analysing the transient response in the terahertz transmission, the charge-carrier dynamics and their properties can be extracted.^12,13^

However, in photoconductors with ultrafast charge-carrier dynamics, non-linearities and influencing factors, such as a dependence on the photoexcitation fluence, can make the extraction of the material properties challenging and reduce their accuracy.^13,23,24^ Here, it is essential to find suitable techniques and strategies as these phenomena may reduce the performance of photoconductive devices during operation.^24^

In this work, we characterise low-temperature-grown GaAs (LT-GaAs) using optical-pump terahertz-probe spectroscopy and terahertz-time domain spectroscopy. An ultrashort lifetime, ranging from 0.4 ps at a pump fluence of 4 µJ cm^−2^ to 0.9 ps at 100 µJ cm^−2^, for the characterised LT-GaAs is demonstrated. We discuss its ultrafast charge-carrier dynamics and how the photoconductivity spectrum is influenced by the charge-carrier dynamics in the context of the ultrafast charge-carrier dynamics of the characterised material.

## Methods

### LT-GaAs growth

A 2 µm-thick layer of LT-GaAs was grown at 210 °C, using molecular beam epitaxy, on a 400-nm-thick sacrificial AlGaAs layer, itself grown on a 650-µm-thick SI-GaAs wafer. After epitaxy, LT-GaAs wafers were coated with the photoresist S1813 for surface protection (no bake) during wafer cleaving. The samples were cleaved into 5 × 5 mm^2^ chips and cleaned in acetone, IPA and DI water under ultrasonication for 5 min, respectively. The LT-GaAs chips were thermally annealed at 550 °C for 15 min in a rapid thermal annealing (RTA) system (temperature ramped up from room temperature to 550 °C in 220 s).

### Sample fabrication

A 10 × 10 mm^2^ sapphire substrate (650 µm thickness) was cleaned by ultrasonication for 5 min in acetone, isopropanol, and DI water, respectively. The sapphire substrate surface was coated with AP3000 as an adhesion promoter and with 1 µm benzocyclobutene (BCB). Both layers were deposited by spin coating. After spin coating, the sample was baked at 150 °C for 3 min.

The cleaved and annealed LT-GaAs chips were dehydrated on a hotplate at 180 °C for 5 min and coated with AP3000 for adhesion promotion. The samples were baked at 100 °C for 60 s and mechanically bonded to the BCB/sapphire substrate in a mechanical bonding jig. A graphite shim was used to evenly distribute the force. The bonded samples were annealed in a vacuum oven at 250 °C for 2 hours while maintaining a force of >500 N cm^−1^. By the bonding and annealing, the BCB thickness is compressed to approx. 100 nm.

After the annealing step, the LT-GaAs was mechanically thinned to 40 µm. For this, the GaAs/BCB/sapphire samples were mounted on a glass block using bonding wax, which was removed after reaching the target thickness with trichloroethylene solvent. The mechanical thinning was followed by chemical thinning in a 4 : 1 vol% citric acid:hydrogen peroxide solution at 40 °C to remove the SI-GaAs until reaching the AlGaAs etch-stop layer. Subsequently, the etch-stop layer has been removed in a buffered HF solution. The target LT-GaAs thickness was 2 µm (area: 5 × 5 mm^2^). The final sample architecture was 2 µm LT-GaAs | 100 nm BCB | 650 µm sapphire substrate (area: 10 × 10 mm^2^).

### Optical-pump terahertz-probe spectroscopy

The sample has been mounted into a vacuum chamber with a pressure of less than 3 × 10^−1^ mbar in order to reduce the superposition of the signal with the resonances from the terahertz absorption by water. All experiments were performed at room temperature. An amplified Ti:sapphire laser (Spectra Physics, MaiTai oscillator seeded into an Empower-pumped Spitfire amplifier) with a wavelength centred at 800 nm (35-fs pulses at a repetition rate of 5 kHz) was used. The beam has been split into three beam paths: (1) terahertz generation, (2) pump beam path for photoexcitation and (3) gate beam for electro-optic sampling. Delay stages for each beam path allow control of the time delay, adjustment of the timing of the three pulses towards each other, and scanning each path individually. For terahertz generation, an anti-reflection and high-reflectivity-coated spintronic terahertz emitter from ref. 25 was used. The used spintronic emitter generates linearly polarised THz radiation with a bandwidth of 7 THz (limited by our detector’s bandwidth). The terahertz beam and the pump beam spots were well aligned to overlap spatially and temporally on the sample position. At the sample position, the terahertz beam width was 0.81 mm (full width half maximum) and the pump beam was 5.15 mm. The terahertz signal was detected *via* electro-optic sampling using a 200 µm (110) GaP crystal. For this, the terahertz beam after sample transmission and the gate beam were focused on the GaP crystal. In electro-optic sampling, a change of polarisation of the gate beam is induced relative to the terahertz intensity.^13^ By directing the gate beam through a *λ*/4 waveplate and a Wollaston prism, the changes in polarisation of the gate beam were detected by balanced photodiodes.^12^ The charge-carrier lifetime was determined by varying pump beam delay at maximum terahertz amplitude and the simultaneous detection of the terahertz intensity relative to the pump time delay. The pump beam fluence was varied with a calibrated filter wheel. Mechanical choppers in the pump beam path and the terahertz beam path, before terahertz generation, were used to collect comparable data with and without photoexcitation by the pump beam. The data was collected *via* custom-built hardware and software.

## Results and discussion

### OPTP measurement

Optical-pump terahertz-probe spectroscopy allows for the investigation of charge-carrier dynamics in semiconductors.^2^ This technique uses the dependence of the transmission in the terahertz spectrum on the number of charge carriers.^23^ This is because the charge-carrier density controls the electrical conductivity of the sample, thereby affecting its absorption of THz radiation.^4,13,26–28^ A higher fraction of the terahertz radiation is absorbed by samples with higher conductivity (metals or metal-like materials) than by those with lower conductivity (insulators or insulator-like materials).^4,13,26–28^ To probe this, OPTP spectroscopy requires a femtosecond infrared (IR) laser for photoexcitation and a terahertz probing pulse.^2,29^ Using a femtosecond IR pulse with an energy higher than the bandgap energy, free charge carriers are generated in the sample.^29^ Subsequently, the time-resolved decay of the change of the terahertz transmission signal −d*T/T* through the sample is measured.^13,30^ This technique provides information on the rate of the recombination of charge carriers and, hence, the charge-carrier lifetime.^13,30^

To study charge-carrier dynamics in LT-GaAs, we conducted OPTP analysis of these samples by photoexciting at 800 nm at different pump fluences. The energy of this wavelength (1.55 eV) lies above the band gap of GaAs (1.43 eV) and, hence, allows for the generation of charge carriers.^29^Fig. 1a shows the terahertz pulse with and without photoexcitation with a pump fluence of 100 µJ cm^−2^ at a fixed pump delay of 900 fs after photoexcitation. It clearly visualises the reduction in the terahertz electric field amplitude when photoexcited compared to the pulse without photoexcitation. The monitored changes in the maximum amplitude of −d*T/T* and the normalised measurement of charge-carrier recombination dynamics for pump fluences between 4 µJ cm^−2^ and 100 µJ cm^−2^ are shown in Fig. 1b and c. Please note that the data in Fig. 1b and c is frequency-averaged and does not allow for the extraction of spectrally resolved information.

**Fig. 1:**
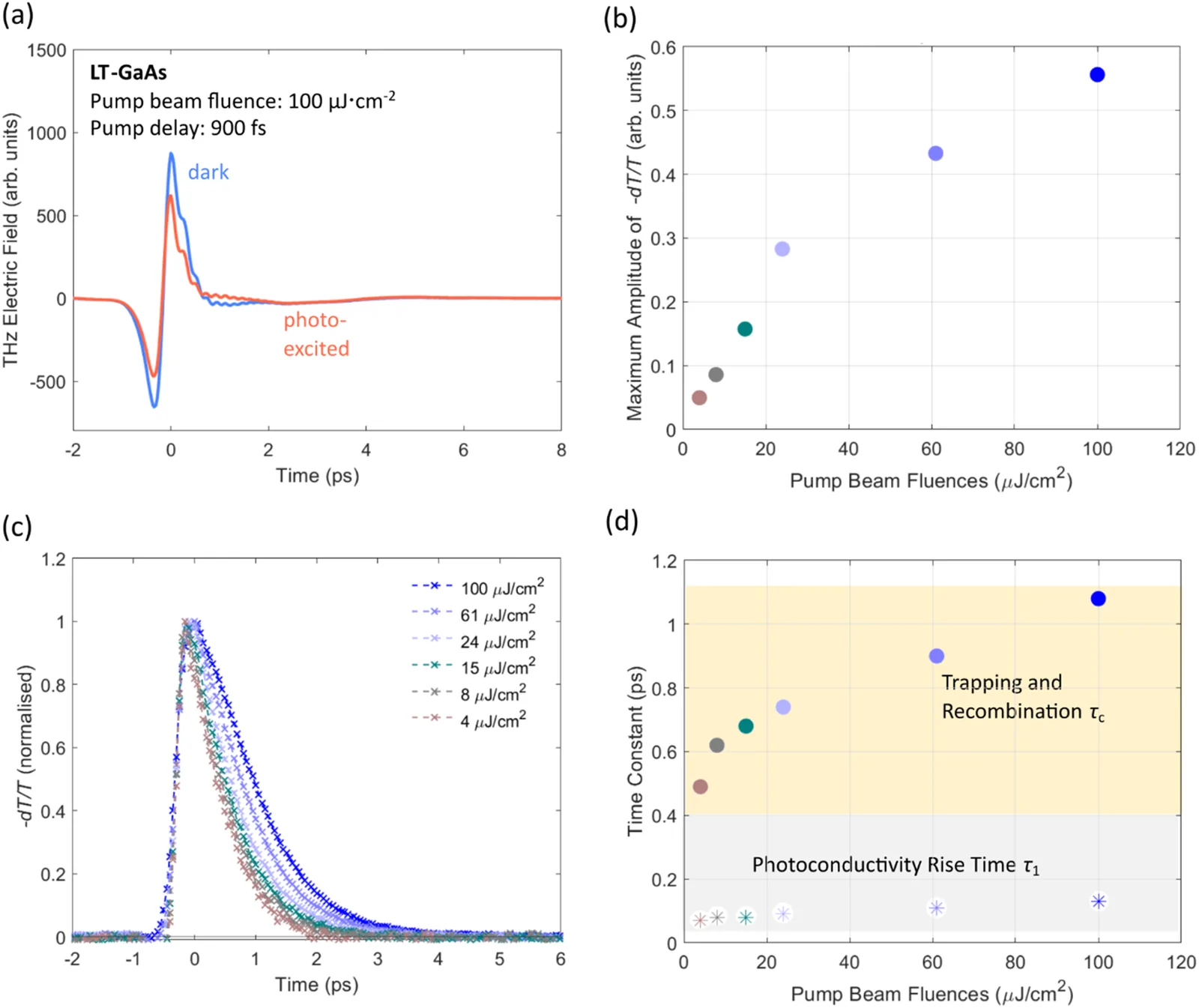
OPTPS measurements of LT-GaAs: (a) transmission measurement with and without a pump beam fluence of 100 µJ cm^−2^ at a fixed pump delay of 900 fs after photoexcitation, (b–d) charge-carrier recombination dynamics of LT-GaAs for pump beam fluences between 4 µJ cm^−2^ and 100 µJ cm^−2^. (b) Maximum amplitude of −d*T/T*, (c) to the maximum peak normalised measurement of −d*T/T* and (d) extracted time constants of the conductivity rise time (increase of −d*T/T*) *τ*_1_ and the effective time constant for trapping and recombination *τ*_c_ (decrease of −d*T/T*).

Fig. 1b demonstrates that higher pump fluences lead to larger changes in terahertz transmission −d*T/T*. This agrees with the increased concentration of generated charge carriers at higher pump fluences.^2,9,27,31^ The maximum amplitude of −d*T/T* only scales linearly for pump beam fluences below 23.7 µJ cm^−2^ (Fig. 1b). For pump fluences above 23.7 µJ cm^−2^, the increase in the maximum amplitude of −d*T/T* does not increase as fast as for lower fluences (Fig. 1b). Based on the literature, the maximum amplitude of −d*T/T* should be relative to the increase in the number of charge carriers, *i.e.*, the conductivity, of the sample.^32^

The fluence-dependent curves in Fig. 1c show a rise in −d*T/T* over a time scale of a total length of 0.3–0.5 ps until reaching the maximum of −d*T/T*, which is followed by a decay of the signal over 1–4 ps before recovering the original state. The required time delay until the signal reaches its maximum is known to originate from state filling.^22^ It is based on the photoexcitation of charge carriers from the valence band to the conduction band.^23^ The subsequent decay in −d*T/T* is determined by trapping and recombination of the photoexcited charge carriers.^32^ Both slopes are influenced by the rates of the individual mechanisms, by diffusion and by depletion of the charge-carriers and trap states.^13,30^Fig. 1c and d shows that the rise of −d*T/T* is similar for all measured fluences, but differences can be seen in the decay.

Since the measurement was conducted on the same sample and only the fluence of the optical pump beam has been varied, the diffusion coefficient should be consistent for the measurements presented in Fig. 1. The pump fluence-dependent time constants of the conductivity rise time have been determined by a monoexponential fit and are summarised in Table 1. The values lie between 0.07 ps for a pump beam fluence of 4 µJ cm^−2^ and 0.13 ps for a pump beam fluence of 100 µJ cm^−2^. It is known that the state filling and diffusion occur within a time frame^22^ of 100 fs, which agrees with the results shown in Fig. 1d and Table 1.

**Table 1:** Evaluated conductivity rise times and charge-carrier lifetimes by OPTP in dependency on pump beam fluence (800 nm, 35 fs-pulses, 5 kHz repetition rate)

Fluence (µJ cm^−2^)	Conductivity rise time *τ*_1_ (ps)	Lifetime trapping and recombination *τ*_c_ (ps)	Calculated defect density *N* (cm^−3^)
99.8	0.13	0.87	3.7 × 10^18^
60.8	0.11	0.77	4.2 × 10^18^
23.7	0.09	0.68	4.8 × 10^18^
15.3	0.08	0.61	5.3 × 10^18^
8.0	0.08	0.54	6.0 × 10^18^
4.3	0.07	0.46	7.1 × 10^18^

The decay, after the maximum −d*T/T* is reached, comes from the charge-carrier trapping and recombination.^33^ For all measured curves, we want to highlight the ultrafast decay of −d*T/T* after the photoexcitation, indicating the ultrashort charge-carrier lifetime (Fig. 1c and d). The charge-carrier lifetime was quantitatively determined by fitting eqn (1) to the decay:^14,34^1
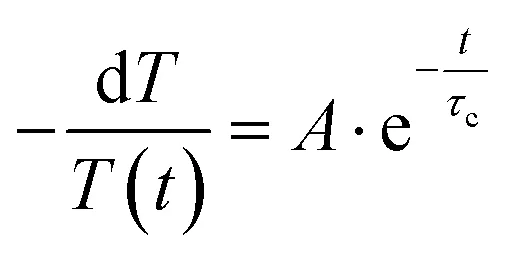
where −d*T/T* is the change in the transmission, *A* is a constant, and *τ*_c_ is the charge-carrier lifetime.

The results, which are visualised in Fig. 1d and listed in Table 1, confirm sub-picosecond charge-carrier lifetimes between 0.46 ps for a pump fluence of 4 µJ cm^−2^ and 0.9 ps for a pump fluence of 100 µJ cm^−2^. We assume that the observed effects solely originate from the LT-GaAs due to the insulating properties of the BCB and sapphire. The determined charge-carrier lifetimes in Table 1 show an increase with increasing pump fluence. This observation clearly suggests a saturation behaviour (Table 1), which will be further analysed in the following.

The charge-carrier lifetime based on photocarrier recombination can be modelled by the Shockley–Read–Hall (SRH) model, as described in eqn (2):^13,17,35^

2 *τ*_c_^−1^ = *N*·*σ*·*v*_th_

where *N* is the defect density, *σ* is the capture cross section and *v*_th_ is the thermal velocity at room temperature.

The thermal velocity can be determined by eqn (3):^13^3
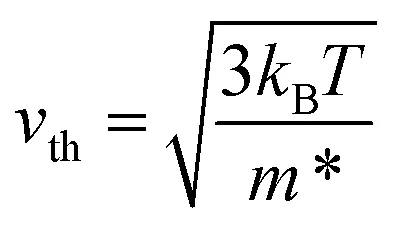
where *k*_B_ = 1.38 × 10^−23^ J K^−1^ is the Boltzmann constant,^36^*T* is the temperature and m* the effective mass of electrons in GaAs.

The electron effective mass for GaAs is 0.063 *m*_e_.^12^ Consequently, *v*_th_ is 4.4 × 10^7^ cm s^−1^. Assuming the same capture cross section of LT-GaAs as in ref. 35 of 7 × 10^−15^ cm^2^, we determined the defect density *N*. The evaluated values are shown in Table 1. It can be seen that a higher defect density is calculated at lower pump fluences (Table 1). Despite the calculated values, the pump fluence does not influence the absolute number of existing defect states in the LT-GaAs, but instead, this observation can be explained by the saturation of trap states as the number of generated charge carriers increases with higher pump fluences:^14,30^

As described in the introduction, the ultrashort charge-carrier lifetime in highly doped semiconductors, such as LT-GaAs, is achieved by the charge-carrier trapping at defect centres.^13,17^ Charge-carrier recombination is slower^37^ than charge-carrier trapping and hence, negligible if the number of photoexcited charge carriers is lower than the number of available trap states per volume. However, if all trap states are filled, recovery of the initial charge-carrier state, *i.e.*, the initial transmission, will be dominated by the slower charge-carrier recombination.^37,38^ In addition, trapping is known to reduce the effective diffusion coefficient due to scattering and, hence, also the averaged charge-carrier mobility.^39^ These mechanisms will lead to an increase in the averaged charge-carrier lifetimes, as observed from Table 1.

### Comparison of the charge-carrier dynamics of LT-GaAs and SI-GaAs

To investigate the charge-carrier dynamics with and without the high concentration of trap centres, we conducted OPTP measurements on the LT-GaAs and on SI-GaAs. Measuring SI-GaAs allows us to selectively test whether the observed properties are related to the ultrashort charge-carrier lifetime. Fig. 2 compares the charge-carrier dynamics of SI-GaAs and LT-GaAs. In Fig. 2a the THz pulse through a SI-GaAs wafer as a background measurement and after photoexcitation with 100 µJ cm^−2^ is presented. The selected pump fluence is the highest pump fluence used in the measurements of the LT-GaAs samples. This pump fluence was selected to achieve the maximum response to the pump beam. The ultrafast decay of the photoconductivity for the LT-GaAs in comparison to the SI-GaAs is clearly visible (Fig. 2).

**Fig. 2:**
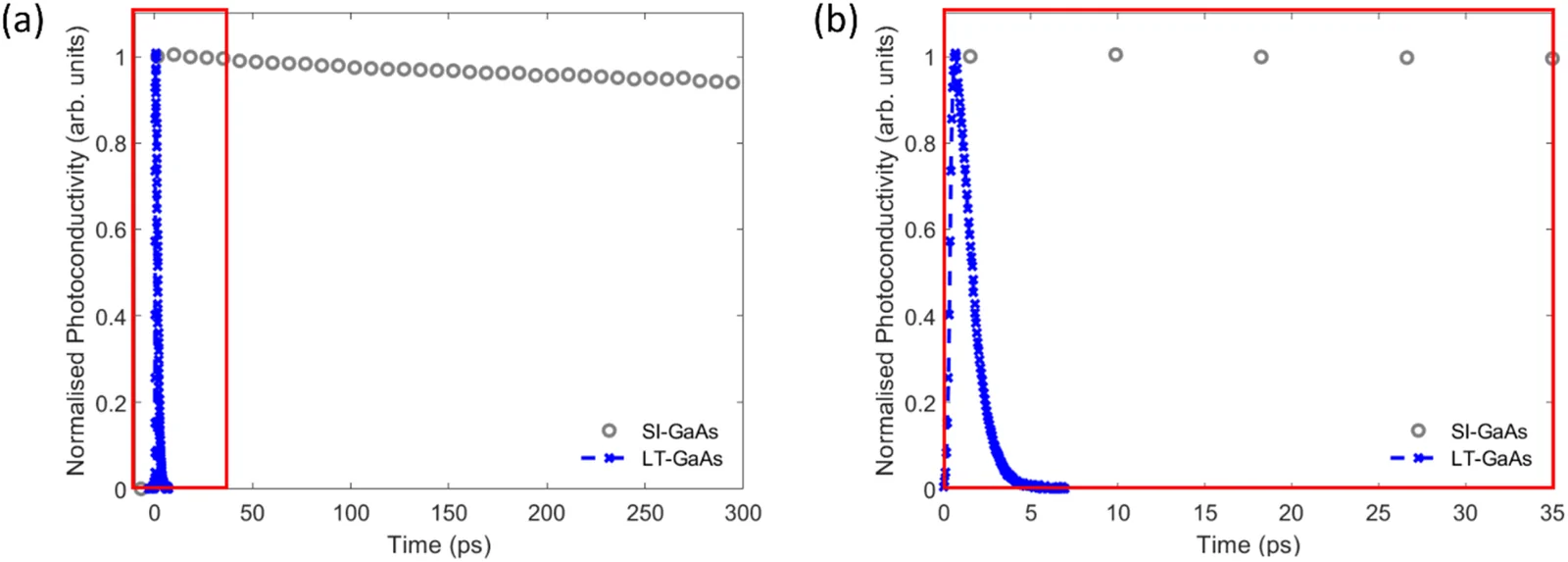
Comparison of the charge-carrier dynamics of SI-GaAs and LT-GaAs at a pump beam fluence of 100 µJ cm^−2^: (a) visualised on the time scale of −10–300 ps after photoexcitation and (b) zoomed in to 0–35 ps after photoexcitation.

In Fig. 3a, the THz pulse through a SI-GaAs wafer without photoexcitation and 25 ps after photoexcitation with a pump beam fluence of 100 µJ cm^−2^ is presented. The pulse shows a significant decrease in transmission by 84% at photoexcitation (pump on) compared to the dark measurements (pump off). As above, this can be explained by the generation of charge carriers by photoexcitation.

**Fig. 3:**
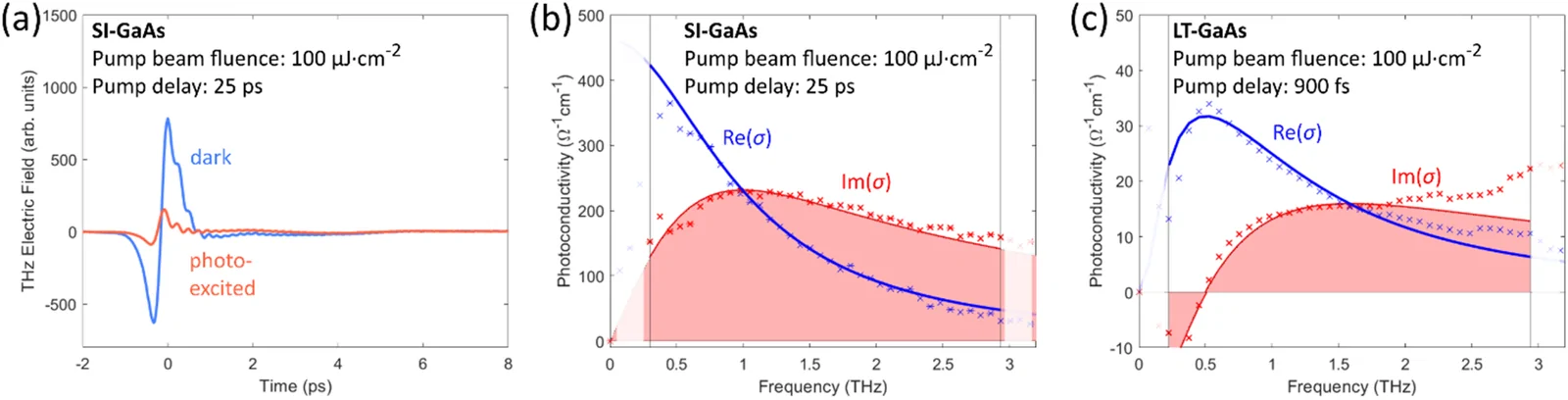
OPTP spectroscopy of SI-GaAs and LT-GaAs: (a) terahertz pulse after transmission through the SI-GaAs sample before and 25 ps after photoexcitation at a pump beam fluence of 100 µJ cm^−2^; and (b) calculated photoconductivity spectrum for a pump time delay of 25 ps. The data has been fitted with the Drude model between 0.3 THz and 3 THz. The extracted charge-carrier density is 6.9 × 10^17^ cm^−3^ and the charge-carrier mobility 4190 cm^2^ V^−1^ s^−1^. (c) Calculated photoconductivity spectrum for a pump time delay of 900 fs for the LT-GaAs sample at a pump beam fluence of 100 µJ cm^−2^. (c) The data has been fitted with the plasmon model between 0.2 THz and 3 THz. The extracted charge-carrier density is 6.8 × 10^16^ cm^−3^, the geometry factor 0.003 and the charge-carrier mobility 2900 cm^2^ V^−1^ s^−1^.

The charge-carrier mobility can be determined by measuring the OPTP photoconductivity spectrum at a chosen time delay after photoexcitation.^12,14,32^ The THz spectrum can be determined by Fast Fourier Transformation (FFT) of the time-domain data. The photoconductivity is evaluated based on eqn (4) for a thin film, considering the dark spectrum compared to the photoexcited spectrum:^31^4
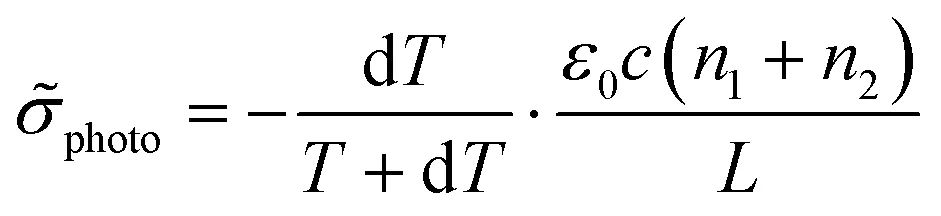
where *σ̃*_photo_ is the photoconductivity, d*T* = *T*_pump-on_ − *T*_pump-off_ is the transient electric-field transmission change, *ε*_0_ is the dielectric constant, *c* is the speed of light, *L* is the thickness of the measured photoconductor (here LT-GaAs), *n*_1_ is the refractive index in vacuum and *n*_2_ is the refractive index of the substrate.^31^Eqn (4) is valid if the substrate below the semiconductor is insulating.^31^

Material parameters of the probed semiconductor, such as the charge-carrier mobility, can be extracted from the photoconductivity by fitting a suitable model.^12^ For thin films and bulk materials, such as wafers, the measured photoconductivity often agrees with the Drude model.^12^ In the case of nanomaterials and micromaterials, an additional depolarisation field has to be considered, which is considered in the plasmon model.^12^ In highly-doped materials or materials with shorter charge-carrier lifetime, further influencing factors and non-linearities on the terahertz photoconductivity spectrum need to be considered.^31^ From the measurement of the SI-GaAs sample, the photoconductivity spectrum has been determined, as shown in Fig. 3b. An agreement of the photoconductivity spectrum of the SI-GaAs sample with the Drude model can be confirmed.

Fig. 3c shows the photoconductivity spectra of LT-GaAs collected 900 fs after photoexcitation with 1.55-eV photons at a fluence of 100 µJ cm^−2^. In contrast to the purely inductive (Drude) response observed from SI-GaAs (Fig. 3b), the photoconductivity spectrum of the LT-GaAs exhibits a resonance at approximately 0.5 THz (Fig. 3c). At frequencies lower than the resonance a capacitive response is observed (Fig. 3c), indicating localisation of charge carriers. The localisation of charge-carriers in LT-GaAs is related to the presence of defects,^15,17^ dislocations^40^ and grain boundaries,^40^ which breaks the perfect periodicity of the GaAs zinc blende^41^ single crystal and creates regions of varying electrostatic potential over length scales of many lattice constants. Hence electrostatic potential barriers and wells exert a restoring force on photo-injected charge carriers, leading to localisation and plasma oscillations.

Charge localisation has been observed previously in photoconductivity spectra of LT-GaAs.^42–44^ In these cases spectra have been modelled in terms of Cole-Cole Drude^44^ and Drude-Smith backscattering^43^ models. Here we fit the data with a Lorentzian function associated with a plasmon model, which has been used previously to describe charge localisation in nano^14^ and microstructured^12^ semiconductors. Details of the model are given in ref. 12.

## Discussion

The measured material and charge-carrier properties of the characterised LT-GaAs are outstanding and allow for high-performance devices for terahertz emission and detection.^10,11^ The reason is that in terahertz setups, the resolution and accuracy of the measurement are highly influenced by the signal-to-noise ratio (SNR) of the used terahertz emitter and detector.^7^ In particular, the emitter performance can lead to significant limitations of the terahertz system.^7^ In photoconductive emitters or photoconductive antennas, the superior properties of LT-GaAs with sub-picosecond charge-carrier lifetime can lead to an improved signal-to-noise ratio (SNR).^11^ For further analysis, we need to look deeper into the device technology and the applied characterisataion technique, and we need to discuss how the measurements can be impacted by the ultrashort charge-carrier lifetime in LT-GaAs.

To reliably fabricate high-performing devices and to further improve the device technology, it is essential to evaluate not only the charge-carrier lifetimes but also their mobility. The above experiments show that the number of generated charge carriers can be tuned by the pump beam fluence, but non-linearities and saturation effects can lead to a non-linear relation between the pump fluence and the number of photoexcited charge carriers and their dynamics. These fluence-influenced dynamics, saturation effects and non-linearities may also vary between material types and by the fabrication technique of the same material, which can make their direct comparison and performance evaluation inaccurate.^37^

Another powerful technique based on terahertz spectroscopy to characterise the charge-carrier concentration in semiconductors is the measurement of the terahertz spectrum after a specific pump delay, as described above. Based on this measurement technique, the photoconductivity spectrum is calculated and can be fitted with a model similar to the Drude model, plasmon model or Drude-Smith model.^12,42,43,45^ For correct parameter extraction, it is essential to select the right model, which is not only material-dependent but also depends on grain sizes or material geometry.^25,31,32,37^ Here in particular, for semiconductors with sub-picosecond charge-carrier lifetime, it is important to know the processes that determine the charge-carrier mobility. Spies *et al.*^32^ names particle–particle scattering, electron–phonon interactions and exciton formation as main mechanisms.

Ulatowski *et al.*^31^ show that, in particular, for materials with sub-picosecond charge-carrier lifetimes, the low dark resistivity needs to be considered in the photoconductivity calculation, and that eqn (4) leads to non-negligible deviations from the exact charge-carrier mobility.^31^ The authors of ref. 31 show higher accuracy of the extracted charge-carrier mobilities and other material parameters when considering the dark photoconductivity. Furthermore, similar to the transient measurements of −d*T/T*, if the photoconductivity spectrum includes resonances, photoconductivity models might not be able to be directly transferred from the same material with a long charge-carrier lifetime, such as SI-GaAs, to materials with sub-picosecond charge-carrier lifetime, such as LT-GaAs. The above equations assume that the optical sample thickness is negligibly small compared to the terahertz wavelength.^31^ LT-GaAs is a highly doped semiconductor, leading to exceptionally short charge-carrier lifetimes, which might instead require consideration of the sample thickness in the photoconductivity calculation.^31^

Additionally, it is important to note that in electro-optic sampling, the pump signal and the terahertz pulse are spatially and temporally aligned to each other.^12^ If the charge-carrier lifetime is in the range of sub-picoseconds, as in LT-GaAs, the signal decay occurs on the same time scale as the length of the THz pulse. Here, Beard *et al.*^44^ mention that deviations from standard models can be observed for pump time delays shorter than 300 fs. This might lead to measurement artefacts and requires additional care in determining and evaluating the respective photoconductivity spectrum.

Summarising, saturation effects, non-linearities in the material response to photoexcitation make the probing of the fluence-dependent charge-carrier dynamics and charge-carrier mobility essential for the development of photoconductive devices. Neglecting these influencing factors can result in reduced signal intensity, bandwidth, and efficiency.^24^ As the charge-carrier dynamics of such advanced materials and thereof-based devices are in the sub-picosecond range, it is important that the measurement equipment has an even faster response time than the dynamics of the sample to be probed, a broad terahertz bandwidth and a high signal-to-noise ratio.^32^ Since terahertz techniques, on the other hand, are also used to develop such new emitters and detectors, which should outperform state-of-the-art devices, this can lead to challenges in the improvement of state-of-the-art techniques.

## Conclusions

In conclusion, we have investigated the exceptional sub-picosecond charge-carrier dynamics of LT-GaAs, which was grown at 210 °C and annealed at 500 °C using rapid thermal annealing, with optical-pump terahertz-probe spectroscopy. This technique allows us to probe the ultrafast charge-carrier dynamics of semiconductors. The samples have been photoexcited under various pump fluences, ranging from 4 µJ cm^−2^ to 100 µJ cm^−2^ with a femtosecond laser with a wavelength of 800 nm. The conductivity rise time constants and the charge-carrier lifetime constants have been determined in dependence on the pump beam fluence. The OPTP analysis proves a charge-carrier lifetime for LT-GaAs at 0.46 ps for a pump fluence of 4 µJ cm^−2^. A saturation effect has been observed. We have discussed that the pump fluence highly influences the charge-carrier density, recovery time and signal-to-noise ratio, leading to non-linearities and saturation effects. The data have been compared to SI-GaAs to visualise the influence of the trap states and the short charge-carrier lifetime in LT-GaAs. We discuss these observations based on the material type and point-out the arising challenges for device fabrication and technology.

Overall, the results are relevant to cutting-edge semiconductors with sub-picosecond lifetimes and to future ultrafast terahertz technology.

## Author contributions

Jasmin-Clara Bürger: data curation (equal); formal analysis (lead); investigation (equal); methodology (lead); visualization (lead); writing – original draft (lead); writing – review & editing (equal). James Normansell: data curation (equal); formal analysis (supporting); investigation (equal); methodology (supporting); writing – review & editing (equal). Lianhe Li: data curation (equal); investigation (equal); methodology (supporting); writing – review & editing (equal). Kun Peng: data curation (supporting); investigation (supporting); methodology (supporting); supervision (supporting); writing – review & editing (equal). Joshua Freeman: conceptualization (equal); funding acquisition (equal); methodology (equal); supervision (equal); writing – review & editing (equal). Michael B Johnston: conceptualization (equal); funding acquisition (equal); methodology (equal); supervision (equal); writing – review & editing (equal).

## Conflicts of interest

There are no conflicts of interest to declare.

## Data Availability

All data supporting the findings of this study are incorporated within the publication.
